# Precision microbial regulation: Strategies for modulating GIT microbiota for host health

**DOI:** 10.1002/imo2.54

**Published:** 2025-01-14

**Authors:** Pei Zhong, Qin Li, Yanmei Zhang, Cheng Guo, Mahmoud M. Abdelsattar, Yanliang Bi

**Affiliations:** ^1^ Institute of Feed Research, Chinese Academy of Agricultural Sciences, Key Laboratory of Feed Biotechnology of the Ministry of Agriculture and Rural Affairs Beijing China; ^2^ Department of Animal and Poultry Production, Faculty of Agriculture South Valley University Qena Egypt

**Keywords:** FMT, genetically engineered microorganisms, gut microbiota, microbial regulation, nanomaterials, phages, synthetic microbial communities

## Abstract

Recent advancements in analytical techniques have unveiled the spatiotemporal diversity of the gastrointestinal tract (GIT) microbiota and their associations with host well‐being. Despite these insights, the precise regulation of GIT microbiota remains a significant challenge. Currently, microbial regulatory strategies, including fecal microbiota transplantation (FMT), synthetic microbial communities (SynComs), genetically engineered microorganisms (GEMs), phages, and nanomaterials, are increasingly utilized for their precise influence on GIT microbiota. This review emphasizes the necessity for developing targeted regulatory strategies in GIT and provides a comprehensive summary and comparison of these approaches to explore their regulatory potential. We discuss recent advancements in these strategies, focusing on their mechanisms, efficacy, safety considerations, clinical trials, and optimization at the application level. Finally, we highlight support methods for optimizing modulation strategies, including the timing of microbial regulation, the selection of microbial targets, and the importance of monitoring the gastrointestinal environment to guide effective microbial interventions.

## Highlights

- Emphasized the necessity of targeted strategies in regulating gastrointestinal tract (GIT) microbiota.
- The precise regulation strategies for GIT microbiota include fecal microbiota transplantation, synthetic microbial communities, genetically engineered microorganisms, phages, and nanomaterials, all of which have been individually reviewed.
- Equipping these strategies with more microbiological discoveries will help improve the precision of selecting intervention timing and microbial biomarkers.

## Graphical abstract

Microbial regulatory strategies such as fecal microbiota transplantation, synthetic microbial communities, genetically engineered microorganisms, phages, and nanomaterials are increasingly being applied for their precise effects on gastrointestinal tract microbiota. This review underscores the need for targeted strategies, discussing mechanisms, advancements, safety, clinical trials, and optimization while highlighting support methods for improving timing, target selection, and monitoring in microbial interventions.

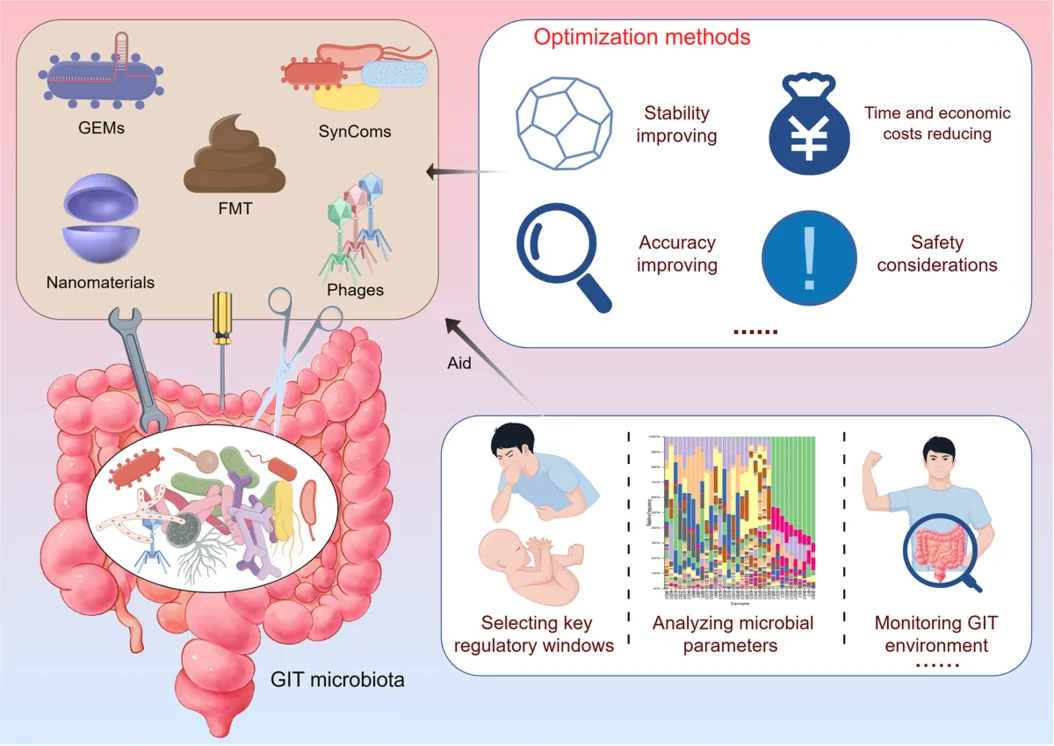

## 1 INTRODUCTION

Microbes establish themselves in the host's gastrointestinal tract (GIT) soon after birth and form a relationship with the host [1]. A stable and balanced GIT microbiota contributes to physiological stability, growth efficiency, and disease control [2, 3]. By applying omics techniques, we gradually decipher the intricate relationship between GIT microbiota and the host's well‐being [4, 5, 6]. This provides a microbiome‐based perspective for understanding the host and paves the way for achieving optimal GIT conditions through selective regulation of its microbiota. Various factors influence GIT microbiota in living organisms [7, 8, 9]. However, in practice, factors such as diet, genetics, and environmental influences are challenging to control comprehensively. Although antibiotics play critical roles in treating diseases, their frequent use can disrupt GIT microbiota, often resulting in microbial dysbiosis [10]. Therefore, probiotics, prebiotics, synbiotics, postbiotics, and antimicrobial peptides (AMPs) have been explored as substitutes. Despite the widespread use of these regulatory strategies, challenges persist regarding their safety and effectiveness [11, 12].

**FIGURE 1:**
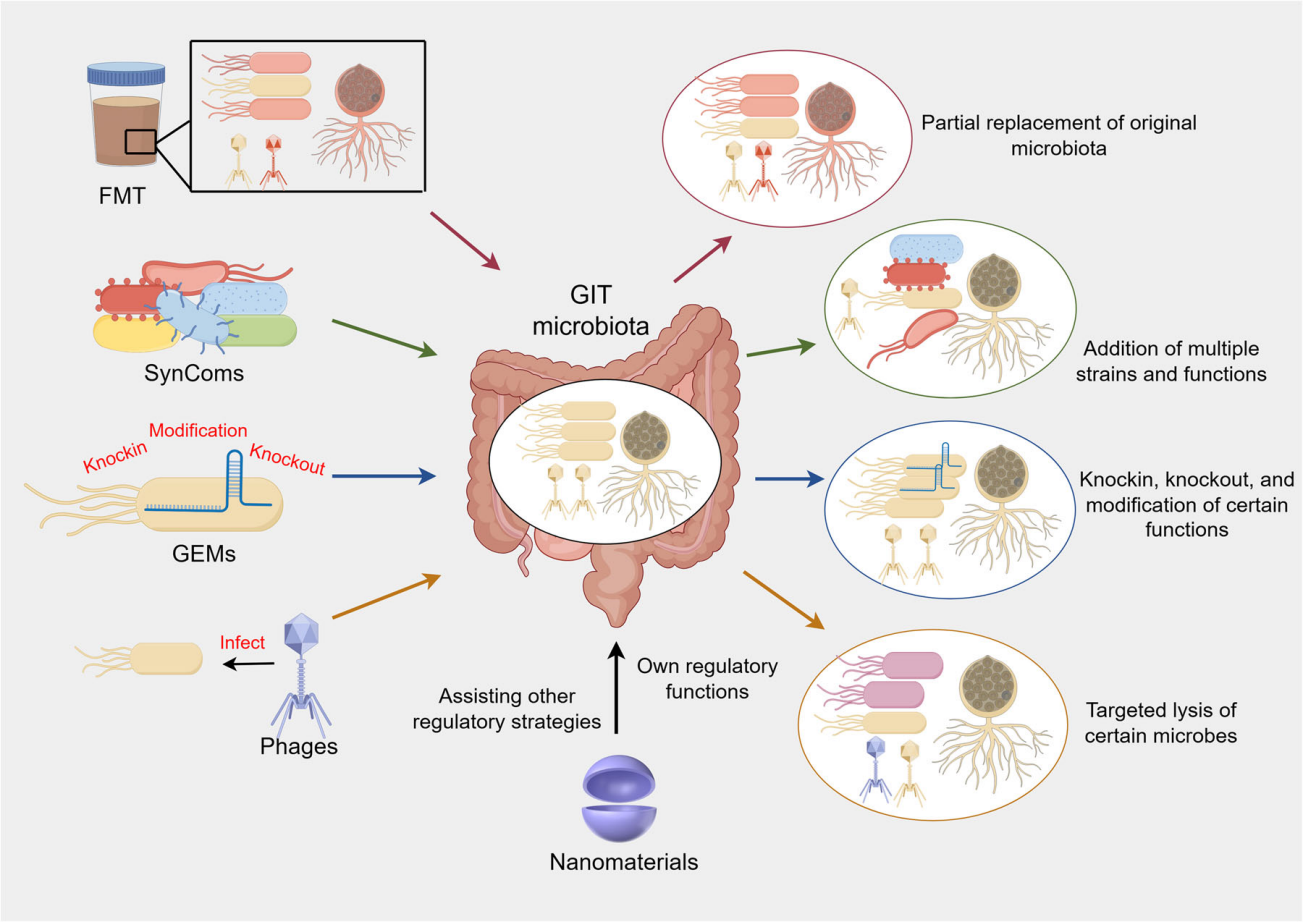
Targeted gastrointestinal tract (GIT) microbial regulatory strategies and their characteristics. Various strategies for targeted GIT microbial regulation each have distinct features. FMT allows bacteria, fungi, viruses, and other microorganisms from donor feces to colonize the recipient's GIT potentially and is commonly used to restore microbial balance. SynComs combine multiple microbes to enhance or restore specific GIT functions. GEMs can insert, delete, or modify genes within the GIT microbiota to perform specific functions. Phages reduce certain microbial populations by targeting and infecting them. Nanomaterials often act as carriers for other regulatory approaches but can also directly influence GIT microbiota. Judicious selection of these strategies can efficiently achieve the desired regulatory outcomes. FMT, fecal microbiota transplantation; GEMs, genetically engineered microorganisms; SynComs, synthetic microbial communities.

Approaches for the targeted GIT microbiota regulation, such as fecal microbiota transplantation (FMT), synthetic microbial communities (SynComs), genetically engineered microorganisms (GEMs), phages, and nanomaterials, have been employed with promising outcomes [13, 14, 15, 16, 17]. Each strategy offers unique advantages in GIT microbiota regulation. For instance, FMT delivers a diverse microbial community, enabling broader and more rapid therapeutic effects [18]. SynComs provides targeted interventions by introducing carefully designed microbial communities [19]. GEMs enable precise genetic modifications for enhanced gut health benefits [20]. Phages offer a direct and efficient approach to pathogen control [21]. However, each has advantages and limitations (Figure 1). Therefore, it is crucial to thoroughly understand their current technological applications and outcomes while remaining vigilant about their potential adverse effects. Furthermore, efforts to optimize these strategies are also a key focus of this review.

Integrating findings from other microbiological studies with targeted microbial modulation strategies may yield better outcomes. Recent discoveries indicate the presence of critical windows for microbial regulation during specific life stages of the host, such as early developmental periods [22]. Additionally, omics‐derived data have uncovered a wealth of microbes associated with host health, and sufficient analysis of microbial parameters can facilitate the selection of key microbial makers for regulation [23]. Moreover, developing methods to monitor the gastrointestinal environment, such as detecting gases to identify abnormal microbial fermentation, will aid in guiding microbial interventions [24]. This review provides a summary and outlook on the advancements of targeted microbial modulation strategies for GIT health, focusing on research findings, application optimization, and microbiological support methods.

## 2 TARGETED REGULATORY STRATEGIES FOR GIT MICROBIOTA

### Necessity for developing targeted regulatory strategies

Current research on GIT microbiota presents both opportunities and challenges. Many studies have noted correlations between specific microorganisms and host well‐being, but these findings need more evidence [25]. Some microbe‐host interactions have been confirmed using single or multi‐microbial colonization methods. For example, by colonizing germ‐free mice with a cocktail of 46 *Clostridium* strains, researchers found that *Clostridium* stimulates intestinal epithelial cells to release TGF‐β and other Treg‐inducing factors, which cooperate with dendritic cells (DCs), leading to a significant accumulation of Tregs in the colon [26]. Colonizing *Candida albicans* in *Card9* knockout mice did not increase systemic antifungal IgG. This led to the further discovery that fungi induce IgG^+^ B cell expansion via CARD9 signaling in CX3CR1^+^ macrophages, resulting in systemic antifungal IgG production [27]. Although these technologies offer partial solutions in elucidating how these specific microorganisms affect the host [28, 29], there are still significant differences between these models and humans. Therefore, developing targeted regulation strategies for GIT microbiota to complement existing methods is valuable for basic research and disease treatment.

Probiotics are widely used in basic research and disease control in GIT [30]. However, some probiotic products exhibit deficiencies in regulating GIT microbiota effectively. Firstly, probiotic functions can be redundant or deficient. For example, various microbial strains‐derived β‐galactosidase can alleviate lactose intolerance when added to dairy products [31]. However, three strains of *Lactobacillus* alone could not treat autoimmune encephalomyelitis in mice, but their mixture inhibited disease progression, highlighting individual strain deficiencies [32]. Secondly, probiotics may not overcome individual differences. Supplementation with 11 common probiotics revealed that original GIT microbes determine the colonization ability of exogenous probiotics [33]. Thirdly, assessing the safety of probiotics is difficult. Although probiotics are used to restore GIT microbiota, they may have side effects on microbial homeostasis restoration, as in some studies, probiotics have been reported as risk factors in disease treatment [34, 35]. Additionally, a meta‐analysis revealed that only 21% of normal individuals experienced a change in microbial structure following probiotic supplementation, highlighting the resistance of GIT microbiota to probiotics and raising questions about their effectiveness [36]. Moreover, probiotics supplementation is insufficient to control other target microbes in GIT precisely [37], which implies that the microbial markers identified through cross‐sectional and cohort studies are difficult to be modulated directly with probiotics.

Currently, targeted regulatory approaches have been tested in clinical trials for various diseases and have shown promising results (Table 1). Thus, these strategies hold potential as next‐generation GIT microbiota modulation techniques. However, a comprehensive review of these approaches is needed to deepen our understanding of microbial utilization, enhance the precision of regulation, and better address human health needs.

**TABLE 1:** Several clinical trials on targeted regulation approaches conducted within the past 7 years.

Disease	Intervention	Outcome	Year	Clinical trial number	Ref.
CDI	FMT (spores)	Reduced the risk of recurrent infections with a good safety profile	2022	NCT03183128	[38]
CDI	FMT	Improved both the physical and psychological condition of the patients	2023	NCT03244644	[39]
SLE	FMT	Improved systemic immune‐inflammation profiles	2022	ChiCTR2000036352	[40]
ASD	FMT	Decreased 5‐HT and GABA levels in serum, and alleviated ASD	2021	ChiCTR1800014745	[41]
CDI	SynCom	High‐dose SynCom prevented the recurrence of CDI	2023	NCT03788434	[42]
CDI	SynCom	Among the 19 patients, 16 (84%) had no recurrence by day 130	2021	NCT02865616	[43]
Advanced Solid tumors	SynCom	Well‐designed SynCom is tolerable and safe in ICI recipients, regardless of tumor type, and may influence the microbiota and metabolites	2023	NCT03686202	[44]
PKU	GEM	Safe and well‐tolerated; a dose‐responsive increase in strain‐specific phenylalanine metabolites was observed	2021	NCT03516487	[45]
PKU	GEM	GEM metabolizes phenylalanine in the gut, reducing postprandial plasma and fasting plasma phenylalanine levels in PKU patients	2023	NCT04534842	[46]
T1D	GEM	The frequency of proinsulin‐specific CD8^+^ T cells decreased	2023	NCT03751007	[47]
Hyperammonemia	GEM	GEM can metabolize ammonia and produce nitrate, leading to a significant dose‐dependent increase in 15 N‐nitrate levels in urine and plasma	2019	NCT03179878	[48]
IBD	Phage	With good safety and tolerance and could survive in GIT	2022	NCT04737876	[21]
GIT issues	Phage	Fecal *E. coli* load was reduced, along with a decrease in IL‐4 levels	2019	NCT03269617	[49]
ICD	Phage	In progress	2021	NCT03808103	[50]
Metabolic syndrome	FVT (FFT)	Influenced the composition of phages in GIT, with good safety and tolerability	2023	NL8289	[51]

Abbreviations: ASD, autism spectrum disorder; CDI, clostridioides difficile infection; IBD, inflammatory bowel disease; ICD, inactive Crohn's disease; PKU, phenylketonuria; SLE, systemic lupus erythematosus; T1D, type 1 diabetes.

### Fecal microbiota transplantation

FMT is a strategy that involves transferring microbiota from a healthy donor into the GIT of another individual [52]. The mechanisms by which FMT affects the host include (1) Reprogramming the GIT microbiota, where bacteria, fungi, archaea, and protozoa from the donor can occupy ecological niches in the recipient's GIT, leading to effects such as immune cell modulation and intestinal barrier repair [53, 54, 55, 56]; (2) Altering microbial metabolites in GIT, such as bacteriocins, short‐chain fatty acids (SCFAs), and secondary bile acids, which can impact other microbes or directly influence host physiology [57, 58]; (3) Affecting host epigenetics through yet unclear mechanisms, such as changes in miRNA expression [59]. For example, researchers found that FMT could restore downregulated miRNAs such as miR‐23a and miR‐150 in mice with recurrent *Clostridioides difficile* infection (rCDI), thereby reducing the expression of interleukin (IL) genes. The combination of miR‐23a and miR‐150 also increased the survival rate of colonic epithelial cells significantly, thus alleviating disease progression [59].

**FIGURE 2:**
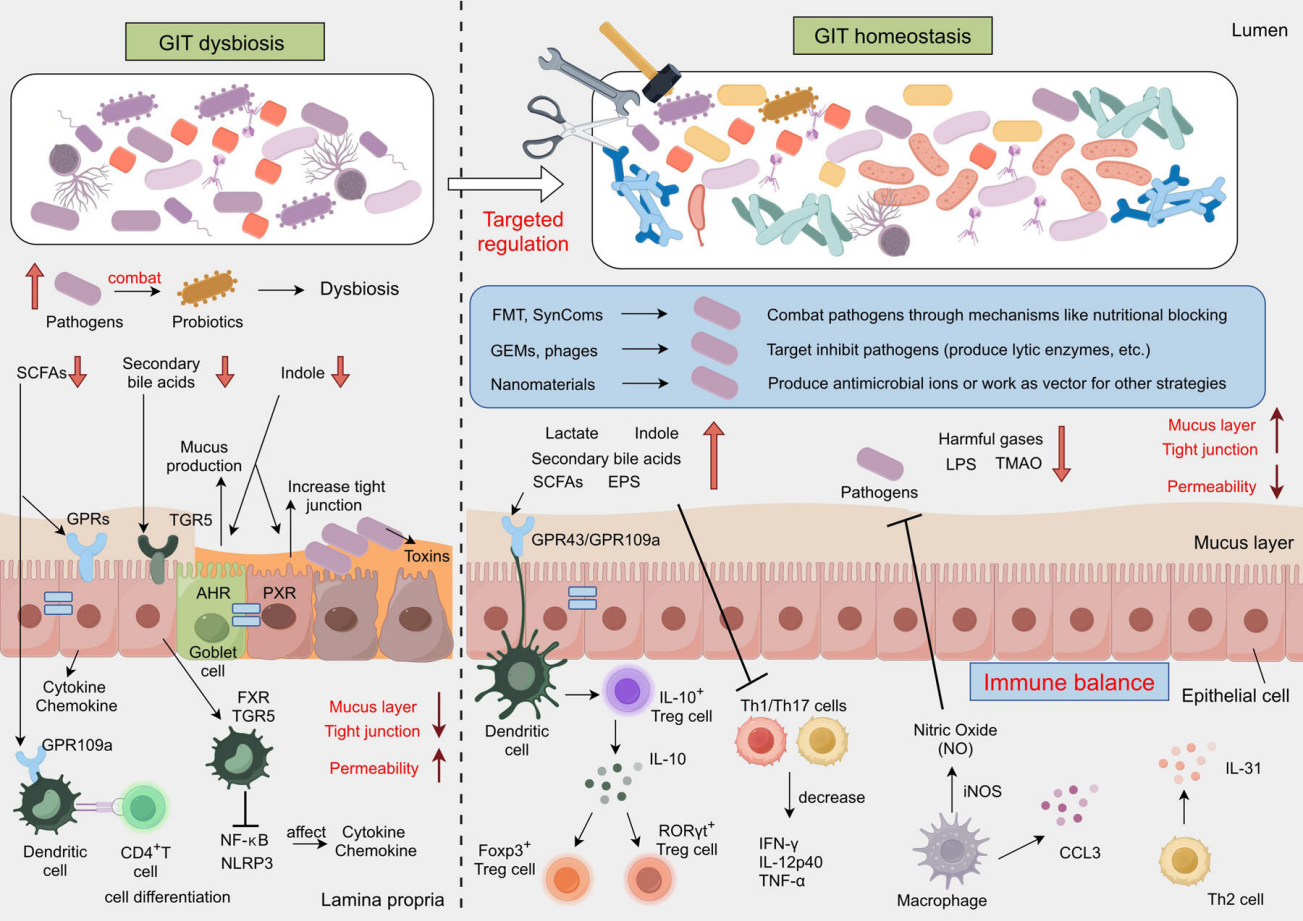
Microbial regulatory strategies can maintain gastrointestinal tract (GIT) homeostasis. Host diseases are often accompanied by GIT dysbiosis. In this state, pathogen overgrowth leads to abnormal microbial fermentation, releasing toxins that disrupt tight junctions and the mucus layer, increasing gut permeability. Abnormal metabolism affects microbial metabolites such as SCFAs and secondary bile acids, which influence immune cells, disrupting gut immune homeostasis. Targeted microbial regulatory strategies can restore GIT homeostasis and metabolism, promoting the balance of the immune system. Specifically, cytokines and chemokines such as IL10, IL31, IL12p40, and CCL3 return to normal expression levels, reducing cellular immune responses and lowering the secretion of inflammatory factors like IFN‐γ and TNF‐α. Additionally, the immune system produces nitric oxide, IgA, and IgG to inhibit pathogens. AHR, aryl hydrocarbon receptor; CCL3, C‐C motif chemokine ligand 3; EPS, exopoly saccharides; FMT, fecal microbiota transplantation; FXR, farnesoid X receptor; GEMs, genetically engineered microorganisms; GPRs, G protein‐coupled receptors; iNOS, inducible nitric oxide synthase; IL, interleukin; IFN‐γ, interferon‐gamma; LPS: lipopolysaccharides; NF‐κB, nuclear factor kappa B; PXR, pregnane X receptor; SynComs, synthetic microbial communities; SCFA, short‐chain fatty acid; TGR5, Takeda G protein‐coupled receptor 5; TMAO, trimethylamine N‐oxide, TNF‐α, tumor necrosis factor‐alpha.

Currently, FMT is a widely used and effective strategy against CDI [60]. Due to its composition of diverse microbes from healthy individuals, FMT has also demonstrated significant efficacy in other situations. It was reported that FMT could replace extended spectrum β‐lactamase (ESBL) producing strains with non‐ESBL strains in human GIT, suggesting that microbial competition in the gut may be a viable strategy for eradicating multi‐antibiotic‐resistant bacteria [61]. The transferred *Odoribacter splanchnicus* strain via FMT increased Foxp3^+^/RORγt^+^ regulatory T cells, induced the secretion of IL‐10, and promoted the production of SCFAs, inhibiting the progression of colitis (Figure 2) [62]. Additionally, an analysis of 1,874 FMT clinical cases from European hospitals revealed that FMT has been successfully used in the clinical treatment of ulcerative colitis, irritable bowel syndrome (IBD), and other diseases [63]. Various factors affect the effectiveness of FMT, and their complexity may limit its broader application in the health industry. Successful FMT requires careful donor selection, personalized design, and appropriate delivery methods, all requiring further research [52, 64]. Analysis of post‐FMT strain dynamics refuted the “super‐donor” hypothesis and emphasized that strain‐level diversity and the complementarity of donor and recipient strains are key to FMT's success [65]. Analysis of FMT outcomes in calves suffering from diarrhea suggested that success may depend on key recipient bacteria of the GIT, such as Veillonellaceae, which facilitate donor microbial colonization [66]. Therefore, scientists advocate for personalized FMT to enhance success rates [67].

Neglecting the safety of FMT could lead to serious consequences, such as the spread of antibiotic‐resistant microorganisms, harmful viruses, and pro‐inflammatory metabolites [68, 69]. FMT can also influence GIT fungi and viruses, leading to uncertain outcomes [54]. A meta‐analysis of FMT failure cases showed that advanced age, medical history, and inpatient status are key predictors of failure. Therefore, assessing patient suitability for FMT is necessary to prevent safety issues from treatment failure [70]. In addition, when administering FMT to immunocompromised patients and children, it is important to prevent potential microbial infections and inflammatory responses [71, 72]. In summary, it is necessary to establish a comprehensive protocol, including donor selection, FMT preparation, and recipient evaluation, enhancing the effectiveness and safety of FMT [73].

### Synthetic microbial communities

The SynComs, comprising various microorganisms, can exert beneficial effects on the host [19, 74]. Unlike FMT, SynComs are well‐defined but incomplete microbial communities. Manipulations such as strain loss, addition, and gene knockout in SynComs, enable precise impact over the GIT microbiota [75, 76]. This allows well‐designed SynComs to influence the host GIT through more flexible mechanisms, including (1) Metabolite produced by designed SynComs, which can be utilized by other beneficial microbes in GIT, ultimately enhancing colonization resistance against pathogens to protect host health [77, 78, 79]; (2) SynComs with high diversity can stabilize GIT microbiota through nutritional blocking, where microbes in the community compete with pathogens for nutrients, thereby limiting pathogen colonization [18]; (3) The diverse microbes in SynComs can work synergistically to achieve tasks, such as inducing the proliferation of beneficial cells (e.g., immune cells), which single microbes alone cannot accomplish [80]. For example, a SynCom composed of 11 bacterial strains could induce the activation of CD103^+^ DCs and major histocompatibility (MHC) class Ia molecules, together stimulating interferon‐γ‐producing CD8 T cells in gut and enhancing the host immune response. This effect was not observed in smaller SynComs [80].

The efficacy of SynComs on host well‐being relies on their design and construction. While SynComs have the advantage of balancing microbial diversity and determinacy, their construction remains complex. From a deterministic perspective, the foundation of building SynComs lies in isolating key strains or enriching specific microbiota. Yet, the pace of culturomics lags behind the discovery rate of metagenomic assembly genomes (MAGs) [81], and current microbiota enrichment techniques lack stability. From a diversity perspective, intricate SynComs can capture the microbial interactions within ecosystems effectively. However, with the growing number of microbial strains in SynComs, it becomes more challenging to maintain the SynComs stability. Therefore, it is essential to construct robust SynComs by exploring mechanisms such as genetic oscillation, synchronized DNA cycles, and biological memory is essential for constructing robust SynComs [82, 83, 84]. Furthermore, including nonbacterial microorganisms, such as viruses, in GIT microbiota also needs consideration [85]. Currently, utilizing tools such as quorum sensing molecules (QS), it is feasible to establish small‐scale SynComs [86, 87]. Two larger GIT SynComs, comprising over 100 strains, have been successfully constructed. They colonized in the intestines of germ‐free mice stably and exhibited functionalities characteristic of normal GIT microbiota [14]. Removing *Clostridium ramosum* from the SynCom SIHUMI and colonizing it in the high‐fat diet (HFD)‐fed germ‐free mice reduced weight gain, showing the flexibility of SynCom applications. This observation prompted further investigation, revealing that *Clostridium ramosum*‐induced obesity can be achieved by enhancing the expression of glucose transporter and fatty acid translocase [88]. In disease control, the SynCom GUT‐108, consisting of 11 strains, influenced GIT microbial metabolism to produce more SCFAs, γ‐aminobutyric acid, and bile acids. This ultimately reduced inflammatory factors such as interferon‐gamma (IFN‐γ), IL‐12p40, and tumor necrosis factor‐alpha (TNF‐α), along with Th‐1 and Th‐17 cells, thereby suppressing IBD effectively (Figure 2) [78].

In conclusion, designing and establishing SynComs for the health industry could be groundbreaking. SynComs research is advancing towards precise design for specific purposes. However, multi‐microbial combinations may pose various unexpected infection risks. Currently, safety testing of SynComs is generally limited to whole‐genome screening of each component to prevent the introduction of antibiotic resistance genes (ARGs) and virulence factors into the host [80]. However, due to the complexity of SynComs, interactions such as competitive exclusion, commensalism, and predation among microbes may lead to unexpected dominant strains or metabolites. Therefore, using SynComs to promote human and animal health requires more stringent safety assessments than those applied to conventional probiotics.

### Genetically engineered microorganisms

Besides FMT and SynComs, GEMs utilize engineering methodologies to modify microbes for human needs by altering existing functions or introducing new ones in GIT [20]. This approach represents a sustainable strategy, particularly considering the slow progress in microbial culturomics. However, these modifications must carefully consider factors such as efficacy, safety, and colonization ability [89, 90]. The mechanisms by which GEMs influence the host are primarily related to the engineered microbial functions, including (1) Integrating biosensors into GEMs with antimicrobial function to detect specific signaling molecules produced by pathogens, thereby enabling targeted disease control [91, 92]; (2) Reprogramming the metabolic pathways of GEMs to produce beneficial metabolites or degrade harmful substances [48, 93]; (3) Enhancing the survival of existing probiotics in GIT by improving traits such as adhesion and stress resistance [94, 95]. GEMs could also have a wider range of functions. An *E. coli* strain was engineered to express phenylalanine ammonia‐lyase from *Arabidopsis thaliana*. This GEM was administered to phenylketonuria mice, resulting in a significant reduction in serum phenylalanine levels [96].

Most GEM studies focus on isolated strains. Considering the limited capabilities of the isolated microbes and their weak adaptation to complex environments [18], in situ modification of GIT microbiota may hold promise as a future research direction. Various techniques were used to construct GEMs, including transformation, conjugation, phage‐based delivery, integrative and conjugative elements (ICE), and others [97]. Using these methods, in situ modification of certain GIT microbiota has been preliminarily achieved [98, 99]. For example, by engineering *Lactococcus lactis* to secrete β‐lactamase within GIT, residual β‐lactam antibiotics were effectively degraded. This strategy prevented the accumulation of ARGs in the gut without altering serum antibiotic concentrations, thereby preserving antibiotic efficacy and mitigating the risk of GIT microbial dysbiosis [100]. Rubin et al. used environmental transformation sequencing (ET‐seq) to identify *Escherichia coli* in infant fecal as a target for in situ genetic editing. Subsequently, they employed a DNA‐editing all‐in‐one RNA‐guided CRISPR‐Cas transposase (DART) system to achieve in situ modifications precisely [101]. Besides enhancing functions, targeted deletion or reduction is also possible. Gene transfer mechanisms have been identified in 88 strains isolated from GIT, with genes successfully manipulated in 27 of them. By deleting the *bai* gene in *Clostridia*, regulation of GIT microbiota and the bile acid pool were achieved, leading to reduced expression of inducible nitric oxide synthase, C–C motif chemokine ligand 3, and IL‐31, and modulation of colitis (Figure 2) [15].

Additionally, discussing the use of GEMs for personalized GIT microbiota regulation requires strict adherence to national regulations and thorough safety assessments of GEMs. Currently, GEMs are primarily used to produce functional products that humans need, such as soy leghemoglobin, carbohydrates, and enzymes [102, 103, 104]. However, utilizing GEMs directly as health supplements to regulate GIT microbiota may still face numerous challenges that need to be addressed.

### Phages

Phage research has been ongoing for a long time. Due to their ability to selectively target specific bacteria and their good safety profile, phages have the potential to serve as an alternative to antibiotics [105, 106]. Phages can replicate through different replication cycles, including lytic, lysogenic, chronic cycle, and pseudolysogeny [107]. Phages influence the host's GIT by many mechanisms, including (1) Phages target and lyse specific microbes, thereby reshaping the microbiota composition [108, 109]; (2) Modulation of host immunity. The host's immune system can detect the presence and activity of phages in GIT, and generally induce an anti‐inflammatory response in the host, such as the production of IFNβ, which contributes to the host's well‐being [110, 111]; (3) Serving as vectors for genetic material, enabling in situ editing of specific microbes in GIT to modify the physiological functions [112].

In recent years, advancements in sequencing depth and the development of bioinformatics tools have led to the discovery of more phages, which are indigenous inhabitants of GIT in humans and animals. Their profound interactions with hosts are elucidated gradually [113, 114, 115]. For example, phages in the adult human GIT were mainly temperate phages that replicate alongside bacteria, driving increased microbial diversity [116]. Phage therapy has been extensively employed in microbiome modulation and treating diseases due to its ability to regulate the GIT microbiota selectively [117, 118, 119, 120]. Phages isolated from sewage could lyse *Enterococcus faecalis* in the mouse GIT in situ, thereby alleviating symptoms of liver disease caused by cytolysin [119]. A phage cocktail of five distinct phages targeted and inhibited antibiotic‐resistant *Klebsiella pneumonia* effectively. This intervention reduced the levels of inflammation‐related immune cells (e.g., IFN‐γ^+^ CD4^+^ T cells) and cytokines (e.g., IL‐15, IL‐17, IL‐9), demonstrating stability and safety within the GIT environment (Figure 2) [21]. The *Escherichia coli* phage øPNJ‐6, isolated from chicken feces, showed long‐lasting antibacterial effects. This effect was attributed to the Hoc protein of phage, which can specifically bind to intestinal mucin [121]. The FVT completely prevented necrotizing enterocolitis (NEC) by reducing the abundance of NEC‐related bacteria in the intestinal lumen of preterm piglets. Compared to FMT, the FVT group did not induce abnormal mucosal immune responses [117]. Additionally, engineered phages can deliver the CRISPR‐Cas system to antibiotic‐resistant bacteria (ARB), achieving targeted lysis of the latter [120]. Researchers engineered λ phage particles with chimeric tail variants to precisely recognize and infect target bacteria, delivering DNA vectors carrying base editors for targeted gene editing. This approach successfully achieved in situ editing of GIT microbiota in live mice [122].

Safety concerns of phages are less studied than bacteria; however, carefully evaluating their impact on humans and animals is still essential. A meta‐analysis of 56 studies found no significant adverse effects associated with phage therapy [106]. However, safety concerns associated with phages should not be underestimated due to the greater diversity and variability of viruses compared to bacteria. Some studies reported that high phage concentrations could induce pro‐inflammatory cytokines, such as IFN‐γ, IL‐12, and TNF, leading to inflammatory responses. Thus, rigorous evaluation of phage dosage is necessary [123, 124]. In the enduring interplay between phages and bacteria, both have evolved intricate mechanisms of mutual antagonism [125, 126]. Therefore, when using phages to regulate GIT microbiota, it is crucial to prevent their misuse to avoid the development of resistance against phages. Phage application should be limited to instances of GIT microbiota imbalance, such as during birth, weaning, diet transitions, and disease, aiming to steer the microbiome toward a healthier state.

### Targeted delivery systems: Nanomaterials example

Advancements in biophysics and biochemistry have significantly enhanced the application of synthetic and natural nanomaterials in microbial regulation. Nanomaterials can serve as carriers for targeted delivery of substances to specific locations for their effects. Their mechanisms as targeted carriers include (1) Passive targeting, where nanomaterials release their encapsulated therapeutic substances at specific GIT sites. For example, pH‐sensitive materials remain stable under acidic conditions and are degraded as the pH increases, enabling drug release in the intestinal environment [127, 128, 129]; (2) Active targeting, where nanomaterials are designed with specific targeting ligands to bind to particular cells or microbial strains in GIT, based on the characteristics of the target [130, 131]. For instance, researchers designed an oxidation‐sensitive ε‐polylysine nanoparticles coated with low‐molecular weight heparin (OPNs@LMWH), which effectively targets inflammatory cells in the colon, thereby reducing levels of reactive oxygen species and pro‐inflammatory cytokines [130].

Nanomaterials enhance the effectiveness of other microbial modulation approaches. Butyrate encapsulated in nanoscale polymeric micelles could be released in different regions of the intestine, increasing the abundance of butyrate‐producing bacteria to reduce the symptom of colitis in mice [132]. Glycosylated nanoparticles, which bind to glucose transport proteins, enabled the targeted delivery of antibiotics to the proximal small intestine, while also protecting microorganisms in other intestinal segments [133]. A polyphenol‐armored nanomedicine transported tannic acid through the stomach and small intestine, thereby specifically targeting inflamed regions of the colon. This approach effectively inhibited the immune response associated with colitis and facilitated the restoration of GIT microbiota [134]. Additionally, the collaboration between nanomaterials and lysozyme could effectively reduce the spread of ARB in livestock production [135].

For the broader application of nanomaterials in practical situations, it is crucial to simplify material preparation to enhance their efficacy [136]. Concurrently, many nanomaterials primarily help reduce pathogenic bacteria, but their effects on other functional microbes need to be further studied to determine their microbial targeting. Additionally, a comprehensive safety assessment of materials is necessary to prevent microbial imbalances and their potential hazards [137]. Certain GIT microbes can directly degrade and metabolize nanomaterials, such as carbon nanomaterials, leading to butyrate production. However, excessive fermentation might disrupt the microbial community, potentially causing dysbiosis [138]. Some studies have also highlighted the potential toxicity of nanoparticles to the mammalian gut. Therefore, stricter considerations are needed when using nanomaterials, including how they are metabolized and excreted by the body after serving their delivery function [139].

## 3 OPTIMIZATION OF TARGETED MODULATION STRATEGIES FOR GIT MICROBIOTA

Laboratory findings on microbiota regulation must undergo rigorous validation through trials to ensure safety and efficacy. Throughout the process, stringent quality control should be implemented, spanning safe manufacturing, distribution, and final delivery to patients and consumers. Therefore, the discussed microbial modulation strategies require optimization to improve precision, enhance stability, ensure safety, and reduce both time and economic costs. These efforts will provide a robust foundation for their eventual widespread application.

### Optimization methods of fecal microbiota transplantation

To enhance the accuracy of FMT for better regulatory effects, researchers have optimized the recipient selection method. Through a meta‐analysis of 24 FMT applications in different diseases, researchers developed a machine‐learning model to predict the most promising donors for shaping the recipient's microbiota, optimizing the donor selection process for FMT [140]. Similarly, Shtossel et al. developed iMic_FMT, a tool that predicts post‐FMT microbiome in human and mouse recipients based on the donor's microbiota composition, further aiding in recipient selection [141]. Since FMT preparations involve numerous microbes, optimizing their stability could directly enhance effectiveness. One approach to improve stability is the cryoprotection of FMT preparations. Researchers used 5% trehalose as a cryoprotectant during freeze‐drying and further encapsulated the FMT preparation with hypromellose capsules. After encapsulation, the microbial cell concentration in each capsule reached 1 × 10^11^. These capsules were later shown to be effective in treating rCDI [142]. The safety of FMT can be enhanced through careful donor screening and optimized delivery methods. Donor screening should include blood tests (e.g., for hepatitis, HIV, and transaminases) and stool tests (e.g., harmful prokaryotic and eukaryotic microbes, viruses, and ARB) [73]. A study explored the effects of rectal administration alone versus oral plus rectal administration in piglets, finding that oral administration may expose the proximal intestine to potential pathogens present in donor feces, leading to adverse effects. In contrast, rectal administration did not result in such outcomes [143]. To minimize the adverse effects of FMT in immunocompromised recipients, the transfer of filtered fecal supernatant was sufficient to relieve symptoms, suggesting that optimizing FMT components represents a promising direction for enhancing the safety of future personalized applications [144]. Additionally, the cost remains a barrier to the broader application of FMT. Optimizing the donor‐selection process by prioritizing lower‐cost tests earlier and postponing those with a lower likelihood of disqualification could reduce the cost per qualified donor by 21.3% [145].

### Optimization methods of synthetic microbial communities

The precision of SynComs microbial construction determines functional accuracy. Two methods are commonly used in constructing SynComs. The bottom‐up approach constructs SynComs by selecting and assembling individual strains with specific functional traits, allowing for precise control over the community composition. In contrast, the top‐down approach simplifies a complex natural microbial community by selectively removing members to retain a minimal effector consortium [146]. For example, scientists successfully constructed a SynCom consisting of 18 strains from an initial set of 37 strains by eliminating strain redundancy, using grouping selection, and performing Spearman rank correlation analysis. This SynCom enabled the decolonization of pathogenic Enterobacteriaceae effectively [147]. The stability of constructed SynComs is crucial for the reproducibility of their effects. A study on a SynCom composed of seven bacterial strains showed that after glycerol preservation and freezing, the microbial structure of frozen aliquots was well maintained, with no significant differences in functionality between fresh SynCom, SynCom frozen for 1 h, and those frozen for 7 days [148]. Additionally, Gnanasekaran et al. demonstrated through in vitro experiments that the initial concentration of strain inoculants does not affect the final stable composition of the in vitro community, providing direction for reducing the economic and time cost of community construction [149]. Using cell models for validation may reduce SynComs' screening time. Researchers constructed SynComs using bacteria isolated from breast milk and validated which SynCom best affects intestinal immunity, barrier function, and apoptosis/proliferation using a quadricellular model of the intestinal epithelium [150]. Our search did not identify a systematic protocol for optimizing the safety of SynComs. However, it is worth noting that even with careful design, SynComs might still increase opportunistic pathogens from the *Escherichia* and *Shigella* genera. This underscores the importance of evaluating the recipient's GIT microbiota, similar to the procedures in FMT [151].

### Optimization methods of genetically engineered microorganisms

The capabilities of GEMs in the field of biosynthesis have seen significant advancements [152]. However, GEM‐based products for regulating GIT microbiota are relatively limited, partly because establishing causal relationships between GIT microorganisms and host health to identify key strains is challenging. Therefore, analyzing microbial parameters to identify essential microorganisms as target strains is crucial for ensuring functional accuracy. Most GIT microorganisms are obligate or facultative anaerobes, which increases the engineering difficulty and may lead to functional loss due to their instability during application. Research on the oxygen tolerance of anaerobic microbes holds promising prospects for their future industrialization. For example, bacterial oxygen tolerance could be gradually enhanced by changing the oxidative stress that microbes may encounter in natural environments (by applying an external voltage to the graphite anode in bioreactors). This enabled them to survive and proliferate in environments with higher oxygen levels [153]. The genetic instability of GEMs may also limit their applications, highlighting the need for developing stable genetic circuits, such as the bistable lambda cI/Cro switch [154]. Government and scientists have imposed strict safety requirements on genetically edited animals and plants and made considerable efforts to enhance safety [155, 156]. However, the safety optimization of GEMs for use in humans is still limited. Therefore, genetic biocontainment systems must be implemented to prevent GEMs from causing harm to humans. These systems should ensure two key aspects: preventing the spread of transgenic material within the host and ensuring that transgenic organisms can survive only within controlled environments [157]. For this purpose, potential strategies include auxotrophic GEMs, toxin‐antitoxin systems, synthetic gene circuits, and horizontal gene transfer (HGT) prevention [158]. Cost optimization for GEMs may be relatively less challenging compared to the improvements above, focusing primarily on optimizing culture media, adjusting environmental factors such as pH and temperature, and recycling and utilizing by‐products [159, 160].

### Optimization methods of phages

There are various optimization strategies for phages. From the perspective of efficacy, phage cocktail therapy, which uses multiple phages in combination, can help prevent the development of bacterial resistance and effectively treat infectious diseases [161]. A continuous arms race exists between phages and bacteria. Further research into phage gene functions and engineered phages can enhance the precision of phage‐based regulation of GIT microbiota. For instance, identifying key proteins that phages use to counter bacterial defense systems will aid in the development of engineered phages [162]. A comprehensive understanding of the target microbes is also required to enhance the precision of phage therapy. Researchers analyzed over 15,000 global *Acinetobacter baumannii* genomes and identified 31 common carbapenem‐resistant strains. Given the geographical homogeneity of these strains, region‐specific phage cocktails were precisely designed and successfully used to treat infections in different areas [163]. To enhance the stability of phages, researchers heat‐treated wild‐type phages at 60°C for 1 h, repeating the process for five cycles, resulting in phages with improved storage adaptability [164]. Additionally, similar to other strategies, cryoprotection and encapsulation of phages can also enhance their stability [165, 166]. The delivery methods of phages also affect their efficacy. For example, researchers used quantitative spray applications for delivery or employed specially designed microparticle carriers to deliver phages [167, 168]. The host range of phages is relatively specific, but swapping tail fibers enables modulation of the host range, unlocking more potential in individual phages. This approach undoubtedly reduces the time and cost required for phage isolation [169]. Given the recognized safety of phages, current safety optimization efforts mainly focus on reducing the dosage to minimize immune responses and decrease phage retention time in the body [170].

## 4 LEVERAGING MICROBIAL KNOWLEDGE TO AID TARGETED MODULATION STRATEGIES

Knowledge of GIT microbiota has made significant advancements in the past decade, and this microbial understanding will aid targeted modulation strategies in better fulfilling their functions. This includes identifying the time windows for microbial regulation, analyzing microbial parameters to determine key microorganisms, and monitoring the GIT environment to indicate microbial abnormalities.

### Windows of microbial regulation

Certain periods in life are key windows for microbial regulation. Early life, dietary transitions, and periods of dysbiosis are critical times when GIT microbiota is particularly responsive to external influences. Identifying and utilizing these windows allows for more precise and effective microbiota‐targeted interventions, promoting long‐term health.

Early life is a window for microbial regulation. The dynamic development of the microbiota after birth and early microbial exposure affects lifelong immune homeostasis [22, 158]. It has been reported that coculturing *Lactobacillus* and *Staphylococcus* with DCs can induce fetal T‐cell proliferation and the release of TNF and IFN‐γ [171]. In a long‐term follow‐up study, individuals with neurodevelopmental disorders (ND) exhibited markedly different infant period GIT microbiota. These early microbial differences might contribute to the long‐term prevention of ND through butyrate turnover and equol production [172]. Additionally, many studies have reported the impact of parental GIT microbiota on the well‐being of offspring, suggesting that the early life window may extend to the gestation period. In male mice with GIT microbiota dysbiosis, the micro‐RNA of their sperm changes. The offspring of these male mice exhibited developmental restrictions, likely due to the impact of male sperm micro‐RNA and hormone level on maternal placental function [173]. The maternal microbiome during pregnancy could influence innate lymphoid cells and F4/80^+^CD11c^+^ mononuclear cells in offspring. This interaction contributes to the immune defense by enhancing the expression of epithelial AMPs [174]. Therefore, taking advantage of this period to perform targeted regulation of the GIT microbiota in parents and infants should be considered for discussion.

Diet changes can stably and rapidly affect GIT microbiota [175]. Poor diets can lead to various diseases by affecting GIT microbiota. Therefore, it is necessary to use targeted microbiota modulation strategies to mitigate their negative effects [176]. Under continual alternation between a HFD and a standard diet, multiple intraspecies mutations were observed in *Bacteroides thetaiotaomicron*. These mutations closely mirrored dietary fluctuations, suggesting that dietary changes can drive the accumulation of microbial genomic mutations, potentially altering microbial functions [177]. High dietary sugars in HFD are also thought to contribute to metabolic disease by decreasing the abundance of *Faecalibaculum rodentium* in GIT, which in turn leads to a reduction in the abundance of segmented filamentous bacteria. These microbial alterations impair the induction of Th17 cells, and ultimately lead to a decrease in intestinal Th17 cell number and function decline [178]. When infants are weaned before 3 months of age, it accelerates the premature acquisition of microbial species and functions in the gut and nasal cavity, leading to a higher risk of asthma in the future [179]. Regulation may be effective during periods of dietary change. For example, adding *Limosilactobacillus fermentum* HNU312 increased the abundance of lipid metabolism‐associated beneficial bacteria, thereby reducing the HFD‐induced fat accumulation [180].

Antibiotic implementation, diseases, and other suboptimal health conditions can easily lead to dysbiosis in GIT. Regulatory strategies can help restore the microbiota and aid in improving host well‐being. For example, postnatal FMT restored dysbiosis in full‐term cesarean‐delivered infants, making their microbial composition more similar to that of vaginally delivered infants [181]. Although antibiotics have cured countless patients, their use often leads to GIT dysbiosis. Researchers found that adding *Bifidobacterium*, particularly strains with higher adhesion capacity, effectively restores antibiotic‐induced GIT dysbiosis [182]. Sub‐health conditions, such as insomnia and anxiety, often lead to GIT dysbiosis. In a mouse model of stress, it had been observed that the diversity of bacteria and viruses significantly differs from that of the control group. FVT has been found to alleviate stress‐related behaviors, and this change is associated with shifts in the abundance of the Caudovirales [183].

### Analysis of microbial parameters: What is best?

Understanding what GIT microbial structures or functions align with human well‐being can better guide targeted modulation strategies. This requires analysis of the microbial parameters. Microbial diversity, core microbiota, enterotypes, and biomarkers such as pathogens or probiotics are commonly associated with host health outcomes. However, researchers need to identify which microbial indicators are effective.

High α‐diversity is often considered to be a more desirable state for the GIT microbiota. For instance, high dairy product intake was associated with increased GIT microbial α‐diversity, which negatively correlates with serum triglyceride levels in humans. Lower serum triglyceride levels may reduce the risk of heart disease [184]. Similarly, infants with higher cognitive abilities exhibit greater GIT microbial α‐diversity, particularly contributed by genera *Bacteroides* and *Streptococcus* [185]. Aging is often accompanied by a decline in GIT microbial α‐diversity and depletion of core groups such as *Bacteroides* [186]. However, livestock with low α‐diversity in their GIT microbiota may exhibit better production traits. The Shannon index of rumen microbiota was lower in the high feed efficiency group, suggesting that lower‐diversity microbial communities may focus more on performing specific functions, such as fiber digestion [187]. However, some studies indicated that host performance is not related to microbial diversity, casting doubt on the effectiveness of using α‐diversity as a key GIT microbial parameter [188, 189].

Since the introduction of the concept of enterotypes [190], their potential to elucidate host traits has become a focus of research. A cohort study identified two clusters of enterotypes, with the *Prevotella copri*‐driven enterotype associated with a lower risk of IBD and better health condition [7]. In another study, patients with an elderly‐specific enriched enterotype were more likely to respond positively to immunotherapy, highlighting the influence of GIT microbiota on tumor therapy [191]. Four enterotypes were identified among people of different ages, with the *Prevotella* cluster predominating in older people and centenarians [192]. Additionally, two enterotype clusters were observed in piglets, with the *Prevotella* and *Mitsuokella* dominated clusters showing significant associations with body weight and postweaning average daily gain [193]. Accurate enterotype identification requires extensive sequencing efforts. The outcomes of enterotype analyses can be influenced by various factors, including host species, experimental conditions, clustering methods, sequencing regions, and OTU calculation strategies [194]. Moreover, enterotypes might overlook functionally important microbes with low abundance [190]. Recently, metabolomics or metabolite detection methods have categorized metabolites into different metabotypes, which are also considered research approaches. For instance, researchers classified patients into distinct metabotypes based on volatile organic compounds and identified metabolic models that accurately predict microbial subtypes, offering insights into biomarkers for IBD [195]. Consequently, enterotypes and metabotypes as microbial indicators warrant further investigation.

Artificial intelligence (AI) algorithms have recently become increasingly popular for analyzing microbial biomarkers that can affect host traits. Machine learning algorithms applied to various groups, including humans, dairy goats, and broilers, identified numerous microbial biomarkers with predictive capabilities for brain development, milk production, and intramuscular fat content [196, 197, 198]. However, the complexity of AI algorithms often overlooks the interpretability of biomarker analyses. Concurrently, with the limitations of microbial culturomics, many studies employing machine learning to analyze microbiome‐trait associations have not yet validated their findings. Furthermore, the reliability of machine learning and deep learning predictions are inextricably tied to the databases, which could impede their generality. This highlights the necessity for more comprehensive research to ascertain their effectiveness.

In conclusion, developing a universally applicable set of microbial parameters to guide targeted modulation strategies remains a considerable challenge. Future research should focus on multi‐omics approaches to identify microbial biomarkers that are closely linked to host traits. Advancing microbial culturomics and developing in situ regulation techniques based on these biomarkers, along with animal models, will be necessary for validating microbial functions. Additionally, engineering strategies aimed at reducing costs will be essential to optimize the application of microbial modulation within the health industry.

### Technological in GIT environment monitoring

**FIGURE 3:**
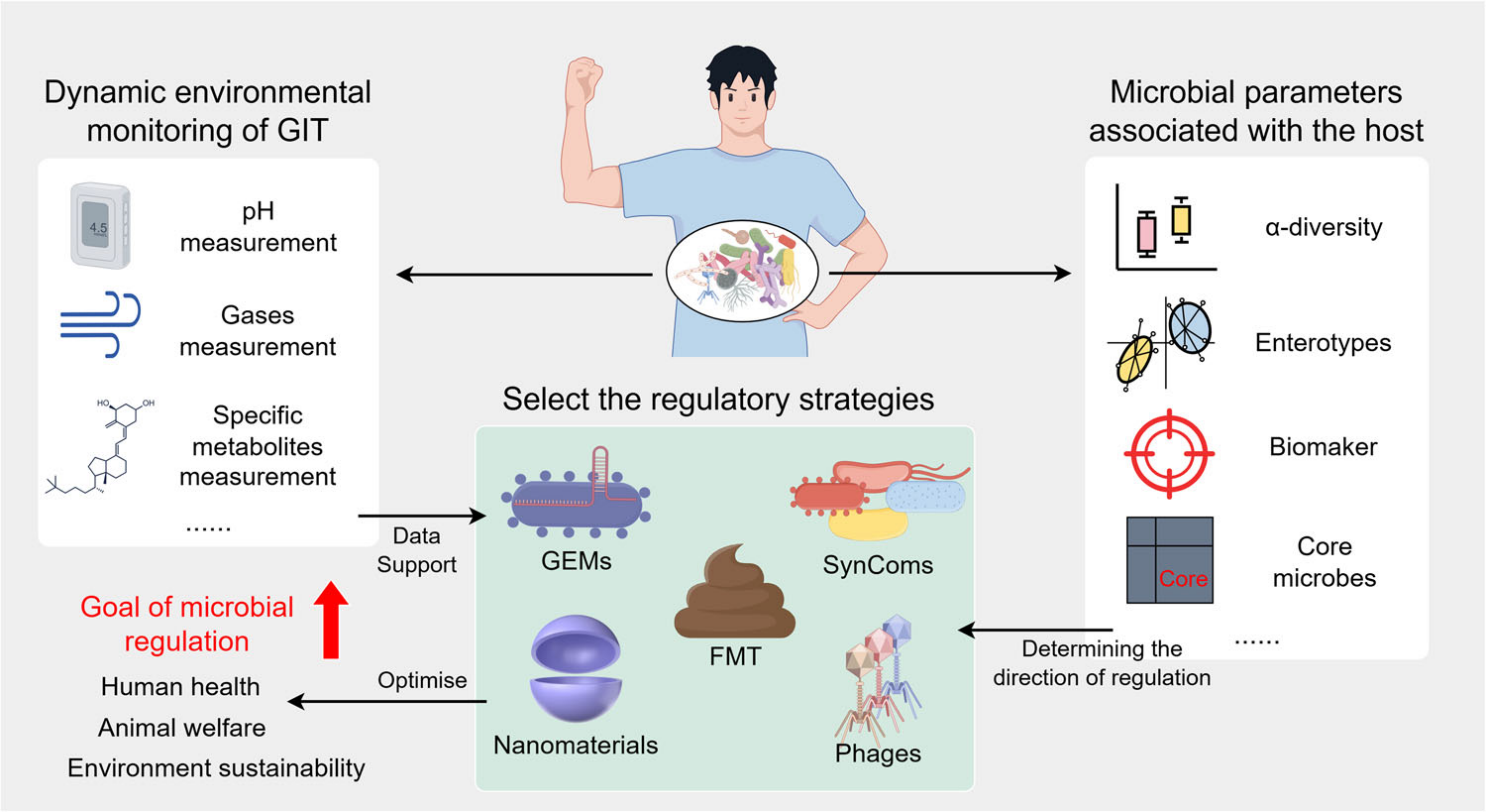
Support methods for regulatory strategies. Monitoring the gastrointestinal tract (GIT) environment is essential for guiding targeted microbial regulation strategies. By closely tracking GIT pH levels, gas composition, and key digestive metabolites, we can detect early signs of abnormal microbial metabolism, such as abrupt pH shifts or excessive gas production. Although parameters like microbial diversity, enterotypes, and the presence of a core microbiota are known to be correlated with various host traits, the precise linkage between these factors and health outcomes remains unclear. This ambiguity can hinder the development and implementation of effective regulatory measures. Consequently, continuous GIT environment monitoring and the precise identification of influential microbial parameters are vital for enhancing host health and ensuring sustainable development. FMT, fecal microbiota transplantation; GEMs, genetically engineered microorganisms; SynComs, synthetic microbial communities.

GIT is a dynamic environment that undergoes long‐term and short‐term microbial fluctuations [199, 200]. Monitoring these fluctuations provides data to support microbial regulation, enabling interventions tailored to specific communities (Figure 3). Using a wireless pH measurement system allowed real‐time monitoring of rumen pH, assisting in the prevention of sub‐acute ruminal acidosis (SARA) [201]. The GIT gases reflect microbial activity. Ingestible electronic capsules could sense O_2_, H_2_, and CO_2_, aiding in the assessment of diet rationality [24]. A GEM, designed based on the principle that inflammation correlates with sulfur metabolism, proliferates when it senses tetrathionate. Therefore, if this GEM is detected in feces, it indicates the presence of GIT inflammation [202]. Researchers integrated genetically modified probiotics into ingestible micro‐bioelectronic devices, successfully monitoring hemoglobin in a gastrointestinal bleeding model of a pig, thereby achieving an accurate diagnosis of gastrointestinal bleeding [203]. Monitoring the GIT environment enables early detection of abnormal fermentation, microbial imbalances, and GIT diseases. This facilitates timely intervention with targeted microbial regulation strategies, preventing treatment delays.

## 5 CONCLUDING PERSPECTIVE

The extensive microbial colonization in the GIT has garnered considerable attention from researchers. Despite numerous challenges, culturomic and metagenomic analyses across various species, life stages, and GIT segments have progressively shed light on the complex GIT ecosystem. Each organism hosts a unique microbiota that plays roles in numerous biological processes and influences host traits.

Targeted strategies for GIT microbiota regulation‐such as FMT, SynComs, GEMs, phages, and nanomaterials‐should be prioritized in research for their potential to address specific health challenges with precision and efficiency. Selecting one or multiple strategies based on specific goals is essential to achieve optimal outcomes. When applying these strategies, it is crucial to consider individual variations to ensure effectiveness and reproducibility while also addressing safety concerns. Future studies must explore fungal, protozoal, and viral communities in the GIT to better understand their interactions with the host, thereby providing a stronger basis for microbial modulation.

Moreover, microbial interventions must be designed with care to prevent adverse effects on the environment and human health. When considering targeted GIT microbial regulation techniques, sequencing technologies can first be employed to assess microbial parameters, informing subsequent interventions within specific microbial regulation windows. Additionally, continuous monitoring of the GIT environment can guide the timing of interventions.

Currently, many promising findings have emerged primarily from animal studies or in vitro experiments, and most causal relationships and mechanisms remain to be fully clarified. Moving forward, scientists should leverage large‐scale data analysis to bridge the gap between research and practical applications in the health industry.

## AUTHOR CONTRIBUTIONS

**Pei Zhong**: Conceptualization; investigation; writing—original draft; writing—review and editing; data curation. **Qin Li**, **Yanmei Zhang**, **Cheng Guo**, and **Mahmoud M. Abdelsattar**: Investigation. **Yanliang Bi**: Conceptualization; funding acquisition; writing—review and editing; resources; supervision.

## CONFLICT OF INTEREST STATEMENT

The authors declare no conflicts of interest.

## ETHICS STATEMENT

No animals or humans were involved in this study.

## Data Availability

Data sharing does not apply to this article as no new data were created or analyzed in this study. Supplementary materials (graphical abstract, slides, videos, Chinese translated version, and update materials) may be found in the online DOI or iMetaOmics http://www.imeta.science/imetaomics/.
